# Functional analysis and transcriptome profile of meninges and skin fibroblasts from human‐aged donors

**DOI:** 10.1111/cpr.13627

**Published:** 2024-02-29

**Authors:** Valentina Fantini, Riccardo Rocco Ferrari, Matteo Bordoni, Eleonora Spampinato, Cecilia Pandini, Annalisa Davin, Valentina Medici, Stella Gagliardi, Antonio Guaita, Orietta Pansarasa, Cristina Cereda, Tino Emanuele Poloni

**Affiliations:** ^1^ Laboratory of Neurobiology and Neurogenetic Golgi‐Cenci Foundation Abbiategrasso Italy; ^2^ Cellular Model and Neuroepigenetics Unit IRCCS Mondino Foundation Pavia Italy; ^3^ Department of Biology and Biotechnology University of Pavia Pavia Italy; ^4^ Molecular Biology and Transcriptomics Unit IRCCS Mondino Foundation Pavia Italy; ^5^ Department of Biosciences University of Milan Milan Italy; ^6^ Department of Neurology and Neuropathology Golgi‐Cenci Foundation Abbiategrasso Italy; ^7^ Center of Functional Genomics and Rare Diseases, Department of Pediatrics Buzzi Children's Hospital Milan Italy; ^8^ Department of Rehabilitation ASP Golgi‐Redaelli Geriatric Hospital Abbiategrasso Italy

## Abstract

The central nervous system (CNS) is surrounded by three membranes called meninges. Specialised fibroblasts, originating from the mesoderm and neural crest, primarily populate the meninges and serve as a binding agent. Our goal was to compare fibroblasts from meninges and skin obtained from the same human‐aged donors, exploring their molecular and cellular characteristics related to CNS functions. We isolated meningeal fibroblasts (MFs) from brain donors and skin fibroblasts (SFs) from the same subjects. A functional analysis was performed measuring cell appearance, metabolic activity, and cellular orientation. We examined fibronectin, serpin H1, β‐III‐tubulin, and nestin through qPCR and immunofluorescence. A whole transcriptome analysis was also performed to characterise the gene expression of MFs and SFs. MFs appeared more rapidly in the post‐tissue processing, while SFs showed an elevated cellular metabolism and a well‐defined cellular orientation. The four markers were mostly similar between the MFs and SFs, except for nestin, more expressed in MFs. Transcriptome analysis reveals significant differences, particularly in cyclic adenosine monophosphate (cAMP) metabolism and response to forskolin, both of which are upregulated in MFs. This study highlights MFs' unique characteristics, including the timing of appearance, metabolic activity, and gene expression patterns, particularly in cAMP metabolism and response to forskolin. These findings contribute to a deeper understanding of non‐neuronal cells' involvement in CNS activities and potentially open avenues for therapeutic exploration.

## Graphical abstract

This work highlights distinct characteristics of meningeal (MFs) and skin (SFs) fibroblasts, derived from 6 subjects with neurocognitive disorders and normal old. MFs exhibit unique timing of appearance, metabolic activity, and gene expression patterns. These findings contribute to a deeper understanding of fibroblasts in CNS activities, for possible therapeutic exploration.

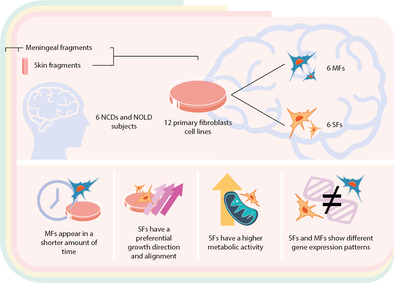

## 1 INTRODUCTION

Meninges are a membrane system composed by three distinct layers with different function: the outermost dura mater, the middle arachnoid, and the innermost pia mater. The dura mater or pachymeninx is the outer layer that envelops the other two layers and the entire central nervous system (CNS), brain, and spinal cord. Both arachnoid and pia mater form the leptomeninges and contain in‐between cerebrospinal fluid (CSF), arteries, and vein.^1^, ^2^, ^3^

The main function of meninges and CSF is the protection of the CNS representing a barrier to the external environment and protecting from trauma.^1^ In addition, the meninges are also present in the deepest areas of the brain located near the choroid plexus, blood vessels, and stroma,^1^, ^3^ as well as, underneath the hippocampal formation.^4^ This distribution and the precise localization suggest a more complex function of the meninges, as a modulator of CNS during homeostatic process and disease.^5^

As well as to act as a barrier, the meninges play a crucial role in corticogenesis and the development of the entire CNS,^6^, ^7^ forming a controlled microenvironment and a well‐organised extracellular matrix (ECM).^8^, ^9^

Experiments on chick embryos have found that the meninges release trophic factors, crucial for the survival of brain cells.^6^ Knockout mice model for CXCL12 and CXCR4 showed that meninges control the neural cell migration and positioning, releasing chemokine CXCL12 through the direct activation by FOXC1.^10^, ^11^, ^12^ Moreover, in Foxc1 mutant mice the release of retinoic acid from the meninges controls the cortical neurons migration.^13^ The structure between brain and pia mater, called pia basement membrane, helps and controls the migration and the positioning of neurons in the cortex. Mice with mutation in the pia basement membrane showed an abnormal distribution of neurons in cerebral cortex and cerebellum.^5^, ^11^, ^14^, ^15^

The meninges are mostly composed of meningeal fibroblasts (MFs), a specialised type of fibroblasts, with a mesoderm and neural crest embryonic origin.^8^, ^16^ MFs have a clear timing of their developmental appearance,^13^ and they are characterised by specific markers, fibronectin, collagen IV, heparan sulphate proteoglycans, and laminin. Nestin and doublecortin (DCX), neuronal markers, were also found in MFs of foetal age rats, forming a neural stem cell niche.^5^, ^17^, ^18^

Advances in MF research, using single‐cell RNA sequencing, have identified several cell subtypes and their functions during rat embryogenesis, emphasising the heterogeneity of meninges and their intricate role in brain homeostasis.^19^

Neural precursor cells, and also cell positive to nestin, have been found in the adult meninges, isolated from mice and rats, both in damaged tissue after ischemia or brain injuries but also in physiological conditions.^2^, ^5^, ^20^, ^21^

DeSisto et al. have demonstrated the conservation of different markers and cell subtypes in both mouse and humans in the foetal age, in particular S100a6 for the pia mater, CRABP2 for the arachnoid and μ‐Crystallin for ceiling cells,^19^ but to date, information and evidence of the human meninges' function and markers in the adulthood are missing.

This work aims to explore and emphasise the peculiarities of MFs investigating the functional characteristics of MFs isolated from aged donors, and their transcriptome profile, compared with skin fibroblasts (SFs), obtained from the same subjects. We evaluated the timing of cell appearance, their metabolic activity, directionality, and the expression markers of characteristic fibroblasts proteins, all compared with SFs derived from the same individual. We also highlighted the difference between the two types of fibroblasts in terms of transcriptome profile, and the differentially expressed genes (DEGs) underlying the up‐regulation in pathways involved in the neuronal differentiation processes, a possible way to generate neurons.

## 2 MATERIALS AND METHODS

### 2.1 Subject recruitment

The population of this study consists of 6 patients enrolled in the Abbiategrasso Brain Bank (ABB) who died from April 2018 until March 2019, with a post‐mortem interval ≤15 h. The peculiar characteristics of the enrolled subjects are reported in Additional file 3: Table S1. We included patients with various major neurocognitive disorders and a control group of normal elderly patients, as characterised by the ABB protocol.^22^ The brain harvesting was approved by the Ethics Committee of the University of Pavia in the context of the InveCe.Ab study (Invecchiamento Cerebrale, ClinicalTrials.gov, NCT01345110), on 6 October 2009 (Committee report 3/2009), in accordance with the principles outlined in the Declaration of Helsinki of 1964 and the subsequent amendments. Brain was explanted and examined at the ABB at the time of death. The male: female ratio is 2 (33.3%): 4 (66.6%). The population's age ranged from 75 to 89 years old (average age of death 82 years).

### 2.2 Cellular isolation from the meningeal and skin tissue

#### 2.2.1 Meningeal and skin fragment dissection

Meningeal fragments (pia mater) of approximately 2–4 cm^2^ were collected during brain harvesting and preserved in sterile phosphate buffer saline (1X PBS) until processed. All samples were processed within 48 h of harvesting. The meningeal fragments were placed in a 10‐cm‐diameter Petri dish and washed twice with 1X PBS (Euroclone, Italy) to remove any residual blood (Additional file 1: Figure S1A). Subsequently, the tissue was separated from the blood vessel (Additional file 1: Figure S1B) and fragmented into small pieces of approximately 2–3 mm using a surgical scalpel (Additional file 1: Figure S1C). One‐two skin fragments were collected from the donor using a surgical punch and preserved in 1X PBS until processed. All samples were processed within 48 h of harvesting. Skin fragments were placed in a 10‐cm‐diameter Petri dish in a complete medium (Dulbecco's modified Eagle's medium (Euroclone, Italy)) at a high glucose concentration containing 20% fetal bovine serum (FBS) (Euroclone, Italy), 2 mM L‐glutamine (Euroclone, Italy), 10 mg/mL streptomycin (Euroclone, Italy), 100 U/mL penicillin (Euroclone, Italy), and 1X nonessential amino acids (Euroclone, Italy). The tissue was fragmented into smaller than 1 mm pieces with a surgical scissor.

#### 2.2.2 Preparation of cell cultures

Meningeal and skin fragments were transferred within 6‐well plates previously treated with 0.5% gelatin (Sigma Aldrich, Italy) for 1 h (Additional file 1: Figure S1D–E). Fragments were cultured and stored in a complete medium with a final addition of 250 ng/mL amphotericin B (Sigma Aldrich, Italy). The plates were then placed in a 5% CO_2_ incubator at 37°C. The culture medium was changed every 3–4 days. Upon reaching 80% confluence, MFs and SFs were treated with 1X trypsin (Euroclone, Italy) to facilitate detachment and subsequent transfer into the T75 flask. When cells reached a confluence greater than 85% (Additional file 1: Figure S1F–G), they were frozen in FBS with 10% dimethyl sulfoxide (DMSO) (Sigma Aldrich, Italy) inside cryovials at −80°C for 24 h, and then in liquid nitrogen. Cells with a passage of no more than 4 were employed for the experiments.

### 2.3 Functional characterisation

#### 2.3.1 Metabolic activity

The MTT assay was used to determine the metabolic activity of MFs and SFs. Therefore, 5 × 10^3^ cells were plated in a 96‐well plate and 5 observation times were identified at day 1, day 4, day 7, day 10, and day 14. The culture medium was removed and the cells were cultured in a 5% CO_2_ incubator at 37°C for 4 h with a 5 mg/mL MTT solution (3‐[4,5‐dimethylthiazol‐2‐yl]‐2,5 diphenyl tetrazolium bromide, Sigma Aldrich, Italy) in FBS‐free complete medium. The MTT solution was withdrawn from the wells and replaced with a 4 mM HCl solution containing 0.1% Nonidet P‐40 (Sigma Aldrich, Italy) in isopropanol (MTT solvent, Sigma Aldrich, Italy). The plate was then incubated for 15 min under agitation and the absorbance was read using a spectrophotometer at 570 nm (EnSight PerkinElmer, USA). The results obtained at various times were normalised on the data obtained on day 1.

#### 2.3.2 Analysis of cell directionality

Cell directionality was measured using the ImageJ Directionality plugin (NIH, USA, https://imagej.nih.gov). Both phase contrast images at 10× magnification (EVOS XL Core Cell Imaging System microscope, Thermo Fisher Scientific, USA) and immunofluorescence images acquired with the fluorescence microscopy at 40× magnification (Axio Imager 2 microscope with Axiocam Mrm camera, Zeiss, Germany) were used to settle the directionality graph.^23^

#### 2.3.3 MACS® separation using magnetic microbeads associated with anti‐fibroblast antibodies

To obtain specific fibroblast cell cultures and to eliminate possible contamination of other cell types after culturing skin and meninges explants, separation was performed using Anti‐Fibroblasts MicroBeads (Miltenyi Biotec, Germany) and Magnetic Activated Cell Sorting (MACS®) (Additional file 1: Figure S1H–I) (Miltenyi Biotec, Germany). Approximately 10 × 10^6^ cells of each cell line considered in this study were centrifuged at 300 *g* for 10 min. The resulting pellet was resuspended in 80 μL of a buffer containing 1X PBS, 0.5% bovine serum albumin (BSA, Sigma Aldrich, Italy), 2 mM ethylenediaminetetraacetic acid (EDTA) (Invitrogen, USA), and 20 μL of Anti‐Fibroblast MicroBeads, and incubated at room temperature (RT) for 30 min. The cells were washed with 1–2 mL of a buffer containing 1X PBS, 0.5% BSA, and 2 mM EDTA and centrifuged again at 300 *g* for 10 min. The supernatant was removed and the pellet was resuspended in the buffer containing 1X PBS, 0.5% BSA, and 2 mM EDTA. Cells were then resuspended and transferred inside an LS column and negative cells to Anti‐Fibroblast MicroBeads were collected. Subsequently, the column was detached from the magnetic separator, and fibroblasts positive for the antibody were extracted using a plunger.

#### 2.3.4 Total RNA extraction

Total RNA was extracted using TRIzol Reagent (Sigma Aldrich, Italy) following manufacturer's instructions. The extracted RNA was quantified using the NanoDrop™ One/OneC Microvolume UV–vis spectrophotometer (Thermo Fisher Scientific, USA), and quality and integrity of the extracted RNA were assessed using the Bioanalyzer 2100 (RNA 6000 Nano Kit, Agilent, Germany).

#### 2.3.5 qPCR

Using human gene sequences available from NCBI (www.ncbi.nlm.nih.gov/nucleotide), PCR oligonucleotide for genes pairs were selected using online Primer3plus (https://www.bioinformatics.nl/cgi-bin/primer3plus/primer3plus.cgi) **(**Additional file 3: Table S2). 500 ng of RNA was reverse transcribed using the iScript™ cDNA Synthesis Kit (BioRad, Italy). Quantitative PCR amplification was performed using SYBR Green Master Mix (BioRad, Italy), and 25 ng cDNA. The reaction was performed using the CFX Connect™ Real‐Time PCR Detection System (BioRad, Italy). Cycle threshold (Ct) values were automatically recorded for each replicate qPCR reaction, and mean Ct values were normalised against those determined for GAPDH. Fold‐expression differences were determined using the 2^−ΔΔCt^ method.

#### 2.3.6 Immunofluorescence assay

MFs and SFs were cultured in 24‐well plates over a coverslip for at least 5 days. The culture medium was removed and the cells were fixed with 4% paraformaldehyde (PFA, Sigma Aldrich, Italy) for 20 minutes at RT. Cells were then permeabilized with 0.3% Triton X‐100 (Sigma Aldrich, Italy) in 1X PBS and incubated for 1 h with 1X PBS with 5% normal goat serum (NGS; Carlo Erba, Italy). Primary antibodies nestin, β‐III‐tubulin, fibronectin, and serpin H1 were then combined and added (Additional file 3: Table S3), and slides were incubated overnight at 4°C. Cells were washed and incubated for 1 hour with secondary antibodies at RT (Additional file 3: Table S3). An additional wash with 1X PBS was then performed. Slides were removed from the wells and mounted on glass slides with a solution containing DAPI (4′,6‐diamidino‐2‐phenylindole, a fluorescent dye capable of binding adenine‐ and thymine‐rich DNA sequences; Sigma Aldrich, Italy), dried and nail‐polished. Analysis was performed using an Axio Imager 2 fluorescence microscope (Zeiss, Germany) equipped with an Axiocam Mrm camera (Zeiss, Germany). The fluorescence intensity was measured using the free ImageJ v.1.54d (W. Rasband, NIH, Bethesda, MD, USA) software (https://imagej.nih.gov/ij/, accessed on September 1, 2023).

### 2.4 Transcriptome profiling

#### 2.4.1 Preparation of libraries for RNA‐seq

Libraries for RNA‐seq from MFs and SFs were prepared with the CORALL Total RNA‐seq Library Prep Kit (Lexogen, Austria) using 500 ng of total RNA, following manufactured instructions. The quality of the libraries was then analysed using the Tape Station (D1000 High sensitivity Kit, Agilent, Germany) and quantified with Qubit™ dsDNA HS Assay Kit (Invitrogen, USA). The sequencing was performed with the NextSeq 500/550 High Output v2.5 kit (150 cycles) produced by Illumina (Illumina, USA).

#### 2.4.2 Bioinformatic data analysis

FastQ files were generated via Illumina bcl2fastq2 starting from raw sequencing reads produced by Illumina NextSeq sequencer (Version 2.17.1.14—http://support.illumina.com/downloads/bcl-2fastq-conversion-software-v217.html). Gene and transcript intensities were computed using STAR/RSEM software using GRCh38 (Gencode Release 27) as a reference, using the “stranded” option. Differential expression analysis for mRNA was performed using R package DESeq.2. Coding and non‐coding genes were considered differentially expressed and retained for further analysis with |log2(MFs/SFs)| ≥1 and a false discovery rate (FDR) ≤0.1, considering SFs as a reference. qPCR was conducted for technical validation of RNA‐seq (Additional file 3: Table S2). The datasets generated and analysed during this study are available in the NCBI GEO repository (GSE255684).

#### 2.4.3 Pathways data analysis and gene ontology

Pathway analysis was performed using KEGG pathway analysis (Kyoto Encyclopedia of Genes and Genomes http://www.genome.ad.jp/KEGG), WikiPathways analysis, and gene ontologies (GO) of differentially expressed coding genes via enrichR web tool (Kuleshov et al. 2016). The R software was used to generate Dotplot graphs (with the ggplot2 library) and GOChord graphs (with the GOplot library). Analysis was conducted by using all the deregulated genes.

### 2.5 Statistical analysis

All parameters and results are shown as mean values ± standard deviation (SD). Statistical analysis was conducted using GraphPad Prism (version 9, GraphPad software), adopting a *t*‐test where indicated. Data with *p*‐values <.05 were considered statistically significant.

## 3 RESULTS

### 3.1 Timing of cell appearance following tissue fragment dissection indicated an expected presence of MFs

**FIGURE 1:**
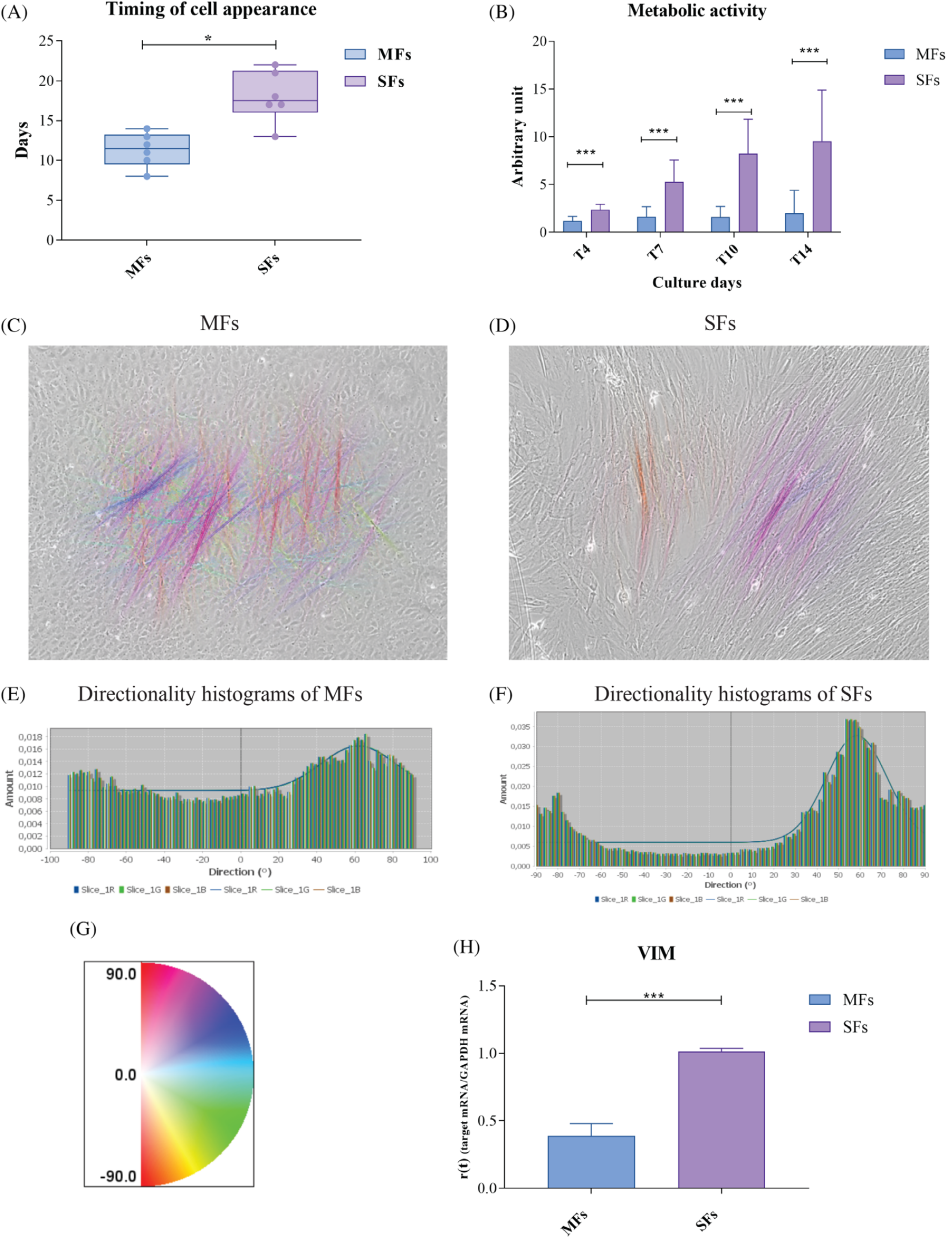
MFs and SFs are functionally two different cell types. (A) Timing of cell appearance of MFs and SFs (*x*‐axis) in terms of days of appearance since the day of explant (*y*‐axis). Black dots indicate MFs; grey dots indicate SFs. Data are shown as mean ± SD (*N* = 6), and analysed using two‐tailed Student's *t*‐test. * = *p* <0.05. (B) Metabolic activity evaluated using the MTT test during 14 days of culture (Cultures Day, *x*‐axis). The observed results were adjusted on day 0 (not reported). During the 14‐day monitoring period, the rate of proliferation of SFs increases. Results are shown as mean ± SD (*N* = 6), and analysed using two‐tailed Student's *t*‐test. *** = *p* <0.001. (C)–(G) Grow direction analysis was obtained using phase‐contrast images using EVOS XL Core Cell Imaging System microscope (Thermo Fisher Scientific, USA) at 10× magnification (C for MFs–D for SFs) and directionality histograms related to MFs (E) and SFs (F). Maps and histograms were obtained using the Directionality plugin from ImageJ (NIH, USA, https://imagej.nih.gov). (G) The rainbow indicates the correspondence of gamma colour of orientation maps (C), (D). (H) Histograms of the VIM expression using qPCR. Results are shown as mean ± SD (*N* = 4), and analysed using two‐tailed Student's *t*‐test. *** = *p* <0.001.

Daily checks were conducted on cultures starting from the day of tissue fragment dissection until 20 or more cells appeared, a threshold set arbitrarily for all cell lines. MFs and SFs exhibit distinct timelines before exiting the tissue (Figure 1A). Specifically, MFs required an average of 11.3 days, while SFs needed 18 days. MFs showed leakage between 8 and 14 days, whereas SFs exhibited leakage between 13 and 22 days.

### 3.2 SFs exhibit higher metabolic activity

The metabolic activity of MFs and SFs was assessed using the MTT assay over a 14‐day culture period, with measurements taken at 4 timepoints beyond time 0 (Figure 1B). From day 4 (T4) to day 14 (T14), SFs consistently displayed higher metabolic activity compared to MFs. Indeed, despite MFs appeared earlier in the culture (Figure 1A), they maintained a relatively lower and stable activity level (Figure 1B).

### 3.3 SFs exhibit a directional growth preference

A distinctive growth pattern concerning cell spatial orientation was observed and analysed to eliminate potential randomness. The evaluation measured the favoured growth direction of MFs and SFs once they surpassed 85% confluence. This assessment utilised phase‐contrast images (Figure 1C, D) and immunofluorescence images labelled with β‐III‐tubulin, a major microtubule component (Additional file 2: Figure S2A, B). The directionality histograms, obtained using the ImageJ Directionality plugin (NIH, USA, https://imagej.nih.gov), display the preferred direction of growth as coloured lines on the phase contrast images (Figure 1C, D), which correspond to gamma colour of orientation maps (Figure 1G). Histograms display on the *x*‐axis the degrees of angle and the number of events on the *y*‐axis (Figure 1E, F). In particular, when it comes to MFs (Figure 1E and Additional file 2: Figure S2C), there is no discernible peak at any given orientation, whereas a preferential growth direction is present in cultures of SFs, as evidenced by the formation of distinguishable peaks at about +60° (Figure 1F) and about −60° (Additional file 2: Figure S2D). This particular orientation and polarisation are due to an upregulation of vimentin, encoded by the *VIM* gene, mainly involved also in the migration and invasion processes.^24^ The gene expression of *VIM* showed a downregulation in MFs (Figure 1H), to indicate a lesser capacity to migrate and to invade the tissue around, explaining the different orientation of these cells, in comparison with well‐organised and aligned SFs cells.

### 3.4 SFs exhibit lower expression of nestin compared with MFs

**FIGURE 2:**
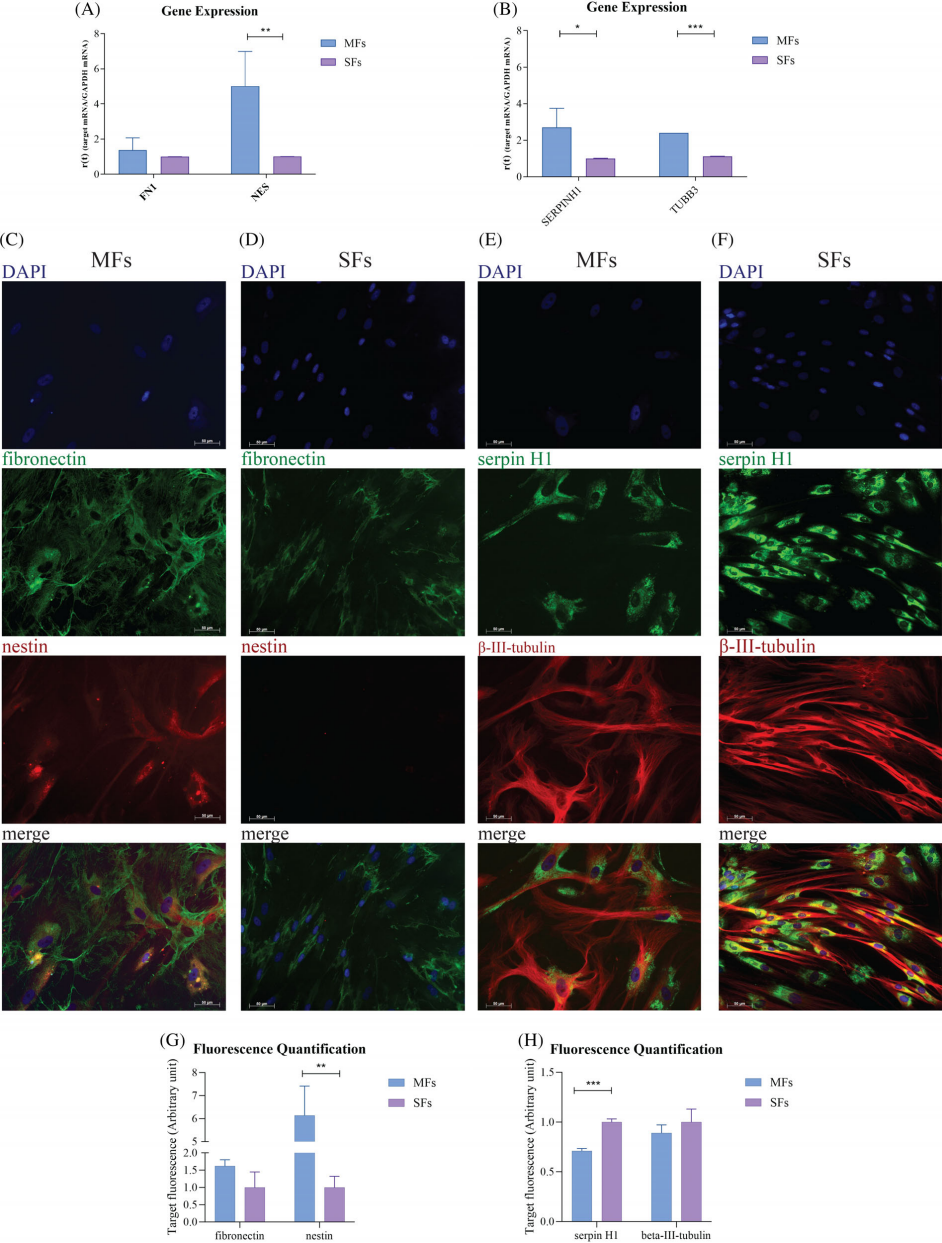
Genes and proteins expression of fibroblasts markers and neuronal markers. (A) Gene expression of *FN1* and *NES* in MF and SFs. (B) Gene expression of *SERPINH1* and *TUBB3* in MF and SFs. Results are shown as mean ± SD (*N* = 4), and analysed using two‐tailed Student's *t*‐test. * = *p* <0.05, ** = *p* <0.01, *** = *p* <0.001. (C)–(F) Immunofluorescence images of MFs (C), (E) and SFs (D), (F) obtained using Axio Imager 2 microscope with Axiocam Mrm camera (Zeiss, Germany) at 40× magnification. (C), (D) In green the fibronectin staining and in red the nestin staining. In blue the DAPI used for staining nuclei. (E), (F) In green the marking with serpin H1 and in red the marking with β‐III‐tubulin. In blue the DAPI used for staining nuclei. (G), (H) ImageJ analysis of fibronectin and nestin fluorescence (G) and serpin H1 and β‐III‐tubulin (H), and analysed using two‐tailed Student's *t*‐test. ** = *p* <0.01, *** = *p* <0.001.

*NES*, *TUBB3*, *SERPINH1*, and *FN1* gene and protein expression was assessed. Nestin, encoded by *NES*, is a marker for neural stem cells; β‐III‐tubulin, encoded by *TUBB3*, is a microtubule element, mainly present in neurons; serpin H1, encoded by *SERPINH1*, is a protein found in the endoplasmic reticulum and a fibroblast marker; *FN1* encodes for fibronectin, a meningeal marker. The comparison between the two cell lines through qPCR analysis (Figure 2A, B) showed that the gene expression related to *NES*, *TUBB3*, and *SERPINH1* is significantly different, and all these genes resulted up‐regulated in MFs (*SERPINH1* * = *p* <0.05, *NES* ** = *p* <0.01, *TUBB3* *** = *p* <0.001). Also, *FN1* tends to be upregulated in MFs, but in a non‐significant manner. The expression of all the above‐mentioned proteins was assessed based on earlier research extracting and defining MFs.^18^ Fibronectin and nestin in immunofluorescence (Figure 2C–H) have the same trend obtained through qPCR in both MFs and SFs. On the other hand, serpin H1 and β‐III‐tubulin showed a higher fluorescence quantification in SFs, respect to the gene expression data. Again, nestin staining showed very low expression in the SFs sample (nestin ** = *p* <0.01, serpin H1 *** = *p* <0.001).

### 3.5 Principal component analysis (PCA) and volcano plot show a different transcriptional profile between MFs and SFs

**FIGURE 3:**
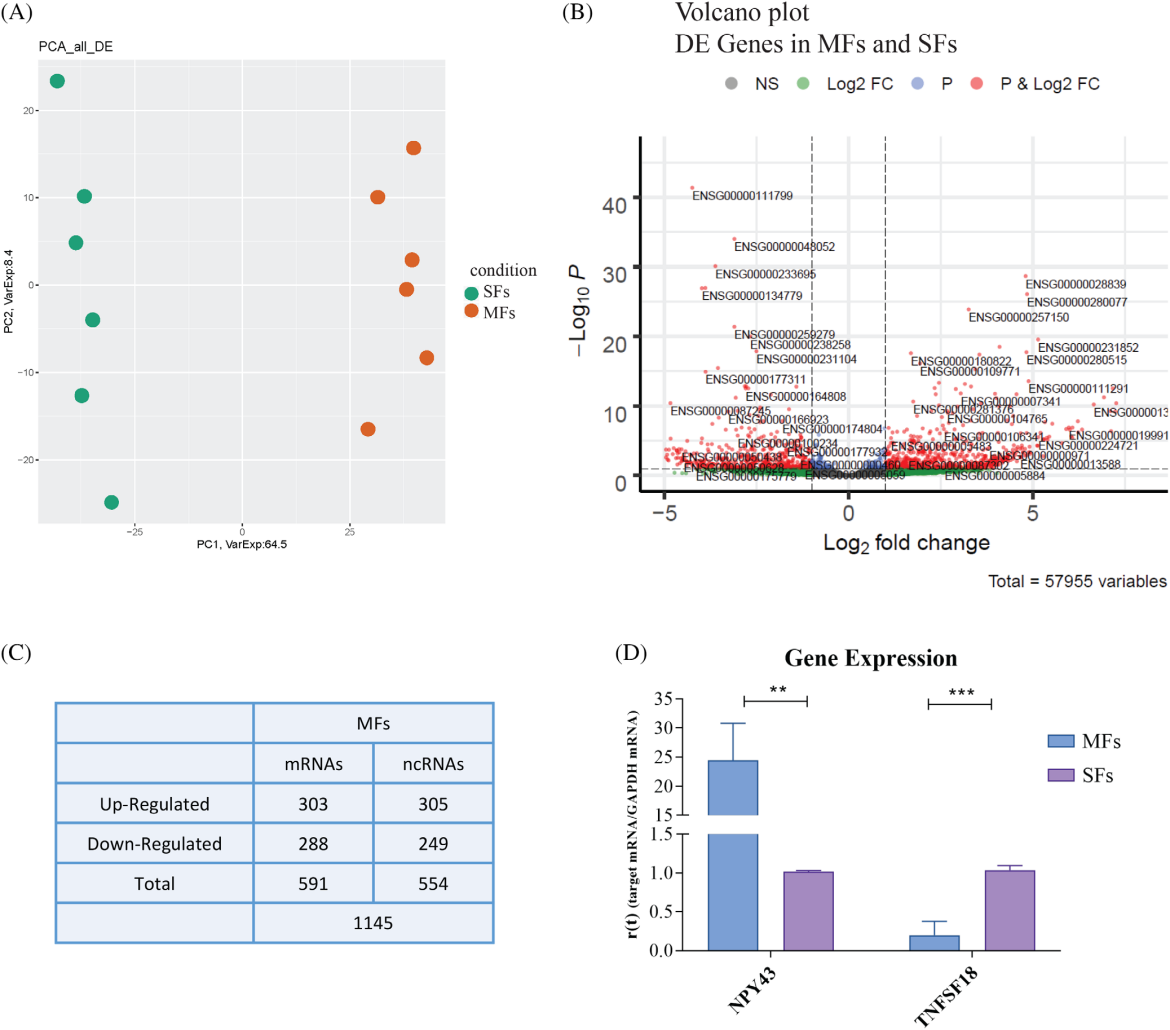
A large deregulation in coding and noncoding genes between MFs and SFs. (A) PCA of DEGs. The dots of different colours indicate the two cell populations (SFs in green and MFs in red). (B) Volcano plot of DEGs between MFs and SFs. Ensemble Gene ID of the most deregulated transcripts are reported, red dots represent differentially expressed genes based on *p*‐value and Fold Change. Only genes with |log2(MFs/SFs)| ≥1 and a false discovery rate (FDR) ≤0.1 were considered as differentially expressed. (C) Table of all DEGs genes divided into up‐regulated, down‐regulated, coding RNA (mRNAs), and noncoding RNA (ncRNAs). (D) Gene expression analysis of *NPY43* and *TNFSF18* in MFs and SFs. Results are shown as mean ± SD (*N* = 4), and analysed using two‐tailed Student's *t*‐test. * = *p* <0.05, ** = *p* <0.01.

To investigate the differences in gene expression and molecular pathways of MFs and SFs, a transcriptome analysis was performed using Anti‐Fibroblasts MicroBeads and Magnetic Activated Cell Sorting (MACS®) to assure a pure fibroblasts cell population. After transcriptomic analysis, a series of de‐regulated genes were identified within MFs (deregulation in terms of |Log2FC|, up‐regulated ≥1, or down‐regulated ≤ −1). PCA of all DEGs showed different expression profiles in MFs compared to SFs (Figure 3A). The volcano plot showed the most significant DEGs in MFs compared to SFs, confirming the different degree of alteration in the two sample types (Figure 3B). A total of 1145 DEGs were identified in MFs, 591 messenger RNA (mRNA), and 554 noncoding RNAs (Figure 3C). The two genes that showed the largest deregulation in MFs compared to SFs in terms of |Log2FC| were taken into consideration among the DEGs identified: qPCR was used to examine the expression of *NPY4R*, the most up‐regulated gene, and *TNFSF18*, the most down‐regulated gene. Because the expression of *NPY4R* is higher in MFs, whereas the expression of *TNFSF18* is higher in SFs, the results obtained by qPCR confirm the bioinformatic analysis of the data (Figure 3D). *NPY4R* is a G‐protein‐coupled receptor that binds the neuropeptide Y (NPY), which has neuroprotective properties.^25^ *TNFSF18*, on the other hand, encodes for the cytokine TNFSF18, which is implicated in immune response modulation.^26^, ^27^, ^28^

### 3.6 Deregulation of ECM‐related pathways in MFs

**FIGURE 4:**
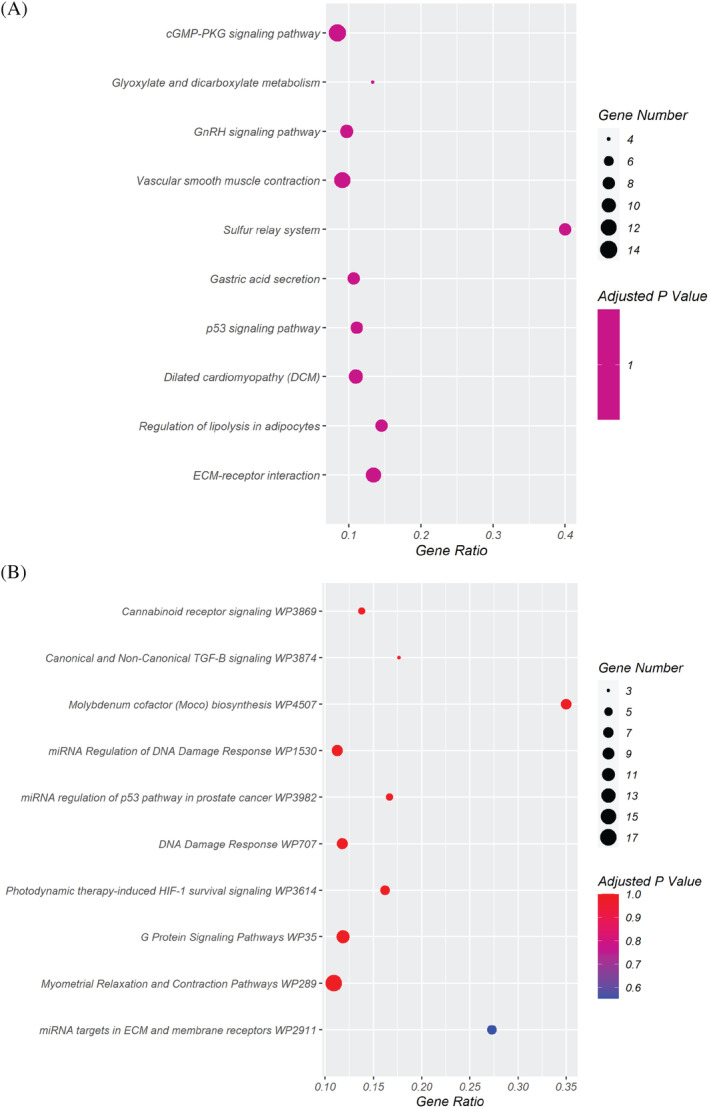
Enrichment analysis from DEGs between MFs and SFs. (A), (B) Dot plot of the top 10 deregulated pathways in MFs according to KEGG analysis and Wikipathways. **A** KEGG analysis. (B) Wikipathways analysis. The *y*‐axis indicates the names of the pathways; the *x*‐axis represents the Rich factor, which is the value indicating the ratio between the number of differentially expressed genes in a given pathway and the total number of genes within the same pathway. The size of the dots represents the number of different genes and the colour indicates the adjusted *p*‐value.

Deregulated transcripts, considering SFs as control, with |log2(MFs/SFs)| ≥1, were subjected to pathways‐related analyses using the EnrichR web tool.^29^ Deregulated pathways were identified using the KEGG 2019 tools (http://www.genome.ad.jp/KEGG) and Wikipathways (https://www.wikipathways.org/) (Figure 4 **).** In particular, the ECM‐receptor interaction results deregulated with the involvement of 11 coding genes. The downregulated resulting genes in this pathway are two types of integrin α (*ITGA4* and *ITGA1*), three types of collagens α (*COL6A1*, *COL6A2*, and *COL6A3*), gene that encode for Thrombospondin‐1, an adhesive glycoprotein that mediates cell‐to‐cell and cell‐to‐matrix interactions (*THBS1*), and gene that encode for tenascin, an ECM protein implicated in guidance of migrating neurons (*TNC*). These genes, correlated to the ECM, are also correlated to the previous result about the cell migration, cell metabolism, and orientation.

### 3.7 Gene ontology enrichment analysis shows the alteration in ECM organisation and in the cAMP metabolism

**FIGURE 5:**
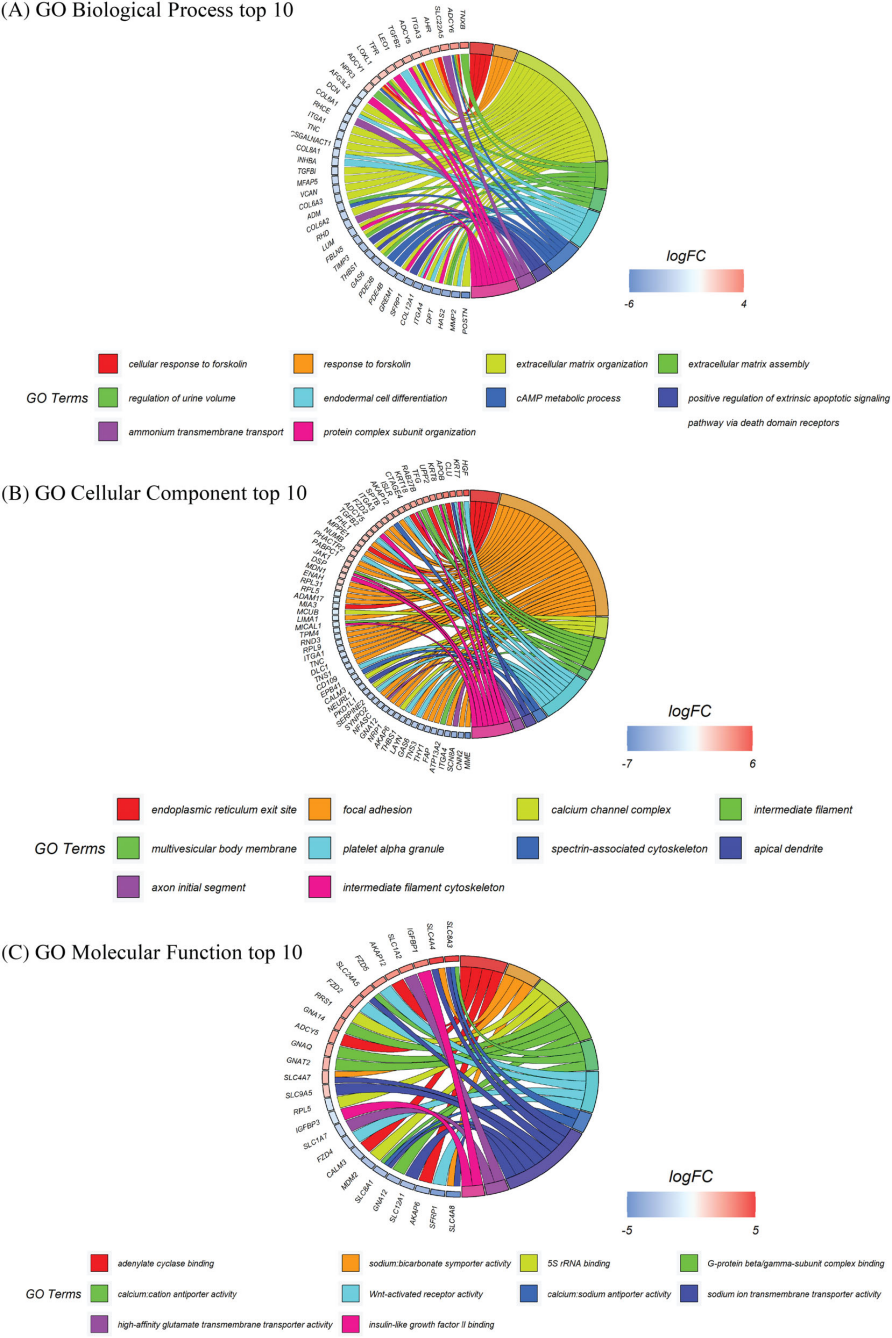
Gene ontology enrichment analysis of DEGs from MFs and SFs. (A)–(C) GO analysis of deregulated genes in MFs. The first 10 GO terms for biological process (A), cellular component (B), and molecular function (C) are shown. Colours refer to different pathways related to deregulated genes. Down‐regulated genes are shown in blue and up‐regulated genes in red.

Expression profiles of deregulated genes related to MFs were subjected to analysis by GO enrichment analysis. Through these analyses, it was possible to define their roles based on biological processes, cellular components, and molecular function (Figure 5). The first 10 GO terms were considered for each category. About biological processes (Figure 5A), there is a majority of down‐regulated genes, in particular the genes involved in the ECM organisation (GO: 0030198) and assembly (GO: 0085029) (Yellow and green in Figure 5B). A minor part of genes deregulated are up‐regulated, and they are involved in two correlated processes: cellular response to forskolin (GO: 1904322), and response to forskolin (GO: 1904321) (Red and orange in Figure 5A), with another process, entirely down‐regulated, strictly correlated to the above‐mentioned process, the cAMP metabolic process (GO: 0046058) (Light blue in Figure 5A). Regarding the cellular components, the majority of deregulations concern the components involved in focal adhesion, a sub‐cellular structure that mechanically links the ECM to cells (GO: 0005925) (Orange in Figure 5B). About molecular functions, a more deregulated process results in adenylate cyclase binding (GO: 0008179), strictly connected to the cAMP metabolic process and also to the forskolin responses (Red in Figure 5C).

### 3.8 Analysis of pathways related to forskolin response show the deregulation of cAMP metabolism

Considering the deregulated biological process, the attention goes to the cAMP because of the results of the unique paper previously published by Colombo et al.^30^ The authors demonstrated that MFs and SFs react differently to exposure to cAMP and astroglial conditioned medium. The expression of genes involved in the biological processes of cAMP metabolism and response to forskolin were analysed using qPCR.

**FIGURE 6:**
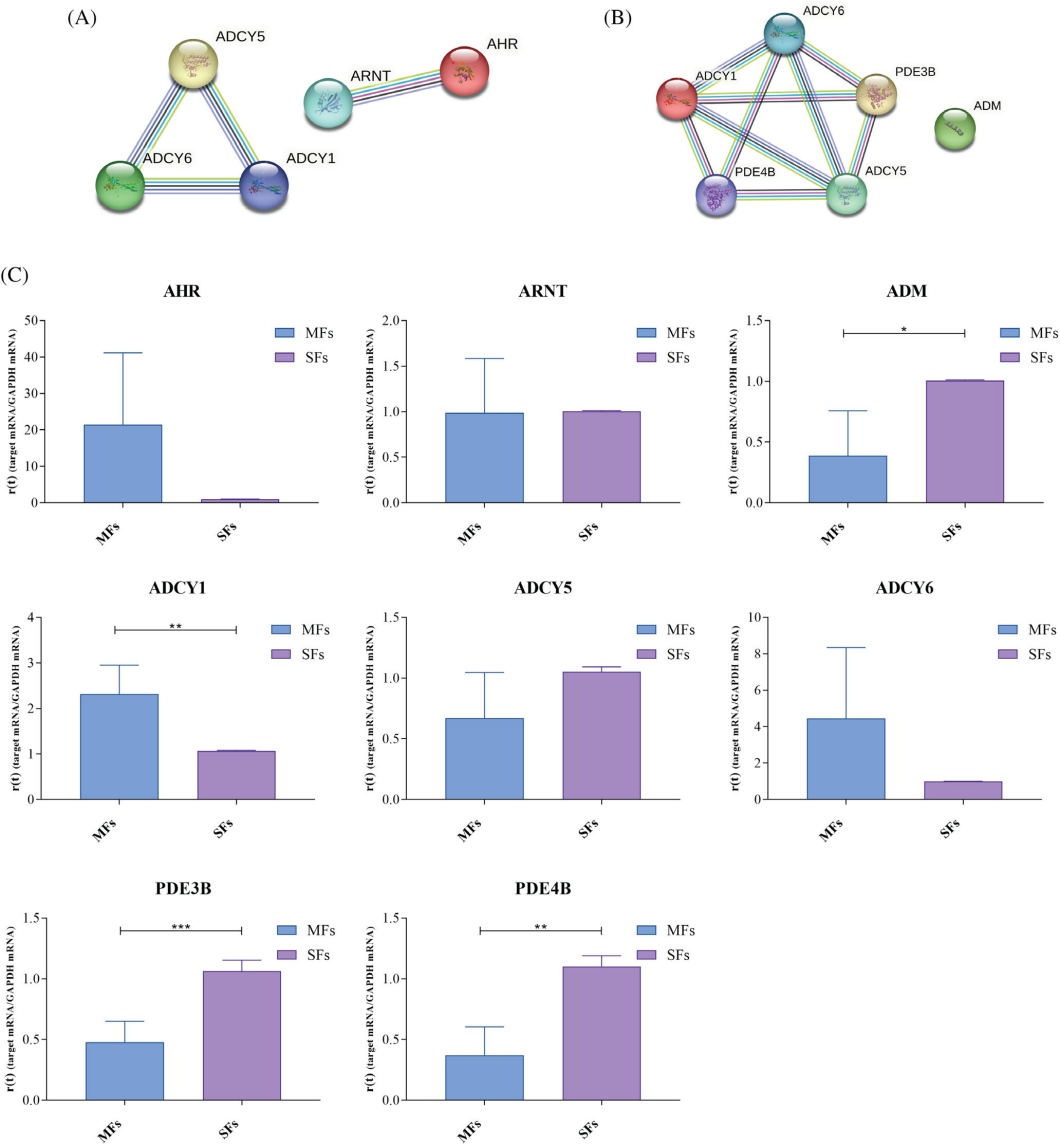
Validation of two deregulated pathways in MFs and SFs. (A), (B) Protein–protein interaction networks obtained by String (https://string‐db.org) related to the processes of cellular response to forskolin (A) and cAMP metabolism (B). (C) Validation by qPCR of gene expression in fibroblasts from MFs versus SFs of the genes involved in both pathways analysed. Specifically, *ADCY1*, *ADCY5*, *ADCY6*, and *AHR* for cellular response to forskolin and *ADCY1*, *ADCY5*, *ADCY6*, *PDE3B*, *PDE4B*, and *ADM* for cAMP metabolism. Gene expression of *ARNT* was also evaluated. Results are shown as mean ± SD (*N* = 4), and analysed using two‐tailed Student's *t*‐test. * = *p* <0.05, ** = *p* <0.01, *** = *p* <0.001.

The pathway inherent to cellular response to forskolin (Figure 6A) includes the involvement of three genes (*ADCY1*, *ADCY5*, and *ADCY6*) encoding for three different isoforms of adenylate cyclase whose expression is increased in MFs (Figure 6C). Within the same pathway, we also find the up‐regulation of *AHR* in MFs, encoding for the aryl hydrocarbon receptor AhR, which heterodimerizes with ARNT (AHR‐nuclear translocator).

Regarding the pathway related to cAMP metabolism (Figure 6B), involvement of *ADCY1*, *ADCY5*, *ADCY6*, *PDE3B*, *PDE4B*, and *ADM* was observed. In MFs, *ADCY1*, *ADCY5*, and *ADCY6* are up‐regulated whereas *PDE3B*, *PDE4B*, and *ADM* are down‐regulated **(**Figure 6C). *PDE3B* and *PDE4B* encode for two different phosphodiesterase isoforms involved in cAMP hydrolysis whereas ADM for adrenomedullin, a peptide with neuroprotective function involved in the cAMP synthesis.

## 4 DISCUSSION

Fibroblasts, the most prevalent and numerous cell types in the body, constitute the majority of the connective tissues. The primary role of fibroblasts is to produce ECM components that keep tissues healthy.^31^, ^32^, ^33^ MFs are necessary for the synthesis of ECM components in both the meninges and the CNS.^34^, ^35^ The majority of MFs produce glial limiting membrane proteins, and they also produce retinoic acid, which is essential for cortical neurogenesis and the development of the cerebrovascular system.^13^, ^36^, ^37^, ^38^

Numerous fibroblast populations as well as neural stem cells have been shown to exist within the meninges. According to all available evidence, this tissue is essential for the CNS's correct development and the capability of cellular regeneration in adult brain parenchyma.^19^, ^20^, ^39^ An in‐depth knowledge of the meninges and the cell types that live within them is necessary to investigate innovative regenerative medicine treatments for neurodegenerative diseases.

Our preliminary conclusions are based on in vitro cell observations to more closely monitor the MFs, cells that are still understudied. The differences between the two cell types, MFs, and SFs, are numerous. First, the cell appearance after explantation from meningeal fragments showed that 10–20 MFs, on average, appeared after 9 days, as previously reported.^18^ SFs, from cultured skin, on the other hand, appeared on average after 17 days. Previously reported data states that SFs shed from cultured skin pieces between 7 and 14 days after explantation, a great difference from our results.^40^, ^41^ It is important to note that both of the explants used in this study were treated postmortem, which may have had effects on the viability of the cells present in the explanted tissues, and a consequent delay in the appearance of the cells. After death, peripheral tissues undergo vasoconstriction, causing faster tissue deterioration. For this reason, our protocol achieved better results by keeping the PMI under 15 h, to avoid cadaveric phenomena that could determine missing or slowed growth of SFs, compared to MFs. Other investigations are needed to elucidate if the anticipated appearance of MFs could be useful for the regenerative processes in brain lesion sites.

We examined the metabolic activity to further characterise the MFs and SFs. Beginning with a similar activity in the first 4 days, the observation significantly diverges during the following 10 days. Despite the older age of the participants may likely have affected both cell types' activity, more in the MFs than in SFs, greater metabolic activity was clearly observed in SFs. This observation was confirmed by previous results, in which SFs are continuously engaged in the ECM rearrangements, with a consequent high metabolic activity.^42^ Since SFs can be easily collected and have a dynamic metabolism that produces energy continuously, they are an excellent model for researching mitochondrial dysfunctions, a critical hallmark for neurodegenerative diseases.^43^, ^44^

After reaching a high level of confluence on a traditional culture plate, without any modifications, SFs demonstrated an organisation's intrinsic capacity. It has been feasible to evaluate the quality of the cell alignment using image analysis, identifying the preferred orientation of SFs and the orientation that is absent in MFs. This could be a result of the intrinsic characteristics of SFs, which are essential for transferring the elasticity and tensile strength that are distinctive to the skin, particularly during the processes of wound healing. The observation that MFs exhibit much reduced metabolic activity and lack an ordered and orientated structure in contrast to SFs is intriguing and somewhat unexpected. When using fibroblasts as a model for neurodegenerative illnesses, it is important to consider the significant functional differences between the two types of fibroblasts, which can be understood in light of this fact. A gene expression analysis showed the downregulation of *VIM*, which could be explained by the different embryonic origins of the tissues they belong to: skin derives from mesoderm,^45^ while meninges derive from elements of head mesenchyme and neural crest.^46^, ^47^ This difference could be explained also using the difference in terms of cell organisation and microtubule compositions.^48^ As a consequence, what we observed seemed to confirm the conclusions of the literature.^48^, ^49^

We investigated the genes encoding fibronectin, β‐III‐tubulin, nestin, and serpin H1 by published studies.^18^, ^50^ *NES* expression is lower in SFs, as well as *TUBB3* and *SERPINH1*. The immunofluorescence analysis showed a very low nestin expression in SFs, compared to MFs, and a higher signal of serpin H1 in SFs. Nestin is a class VI intermediate filament protein expressed in the developing and adult brain, in particular in the mitotically active areas.^51^ Similar to neural stem cells, these nestin‐positive cells could be cultured and developed in active cortical neurons.^52^ Nestin expression in MFs isolated from post‐mortem meninges confirms previous data, adding a possible second function of MFs as a source of new neurons, like neural stem cells, that express high levels of nestin protein, forming a stem cell niche. Additionally, we cannot rule out the possibility that nestin production enables MFs to develop into neurons in the context of brain parenchymal injury to carry out a repair process, actuating a regenerative process, much as how SFs differentiate into myofibroblasts during wound healing.^53^

The gene expression investigations and whole transcriptome analysis provided a more thorough explanation of the distinctions between MFs and SFs. We focused on the genes and functional pathways that have higher MFs than SFs‐specific dysregulation. A significant number of DEGs genes (1145 genes), both coding and non‐coding, was discovered by RNA‐sequencing analysis, confirming all previously documented discrepancies and demonstrating in further detail that MFs and SFs are two fundamentally different cell types.

According to Colombo et al., dysregulation of cAMP‐dependent pathways has attracted a lot of attention in terms of functional pathways.^30^ Gene expression studies have shown that the *AHR*, *ADCY1*, *ADCY5*, and *ADCY6* genes are dysregulated and are implicated in biological processes connected to the response to forskolin. The aryl hydrocarbon receptor AhR heterodimerizes with the AHR‐nuclear translocator and translocates within the nucleus to operate as a transcription factor for genes encoding retinoic acid metabolism, cell proliferation, and differentiation.^54^ The adenylate cyclase genes *ADCY1*, *ADCY5*, and *ADCY6* generate various enzyme isoforms. These genes may be crucial for maintaining a healthy CNS homeostasis because their downregulation or mutation has been associated with different neurological disorders.^55^, ^56^, ^57^ The overexpression of genes linked to the cellular response to forskolin in MFs as compared to SFs may suggest increased susceptibility to cAMP as well as increased susceptibility to forskolin. According to Insel and colleagues,^58^ forskolin, a diterpene produced from *Plectranthus barbatus*, can activate adenylate cyclase and increase cAMP levels, which promotes the differentiation of neural progenitor cells.^59^ This evidence, along with the high expression of nestin, could point to a propensity for reprogramming of this cell type since forskolin and other small molecules are used to stimulate direct reprogramming processes and the transdifferentiation of fibroblasts into neurons.^30^, ^55^, ^60^

The second most deregulated pathway in MFs is the cAMP metabolism pathway. While *PDE3B*, *PDE4B*, and *ADM* were found to be down‐regulated, *ADCY1*, *ADCY5*, and *ADCY6* were found to be up‐regulated. Two phosphodiesterase isoforms, *PDE3B* and *PDE4B*, are involved in the hydrolysis of cAMP.^61^, ^62^ *PDE3B* and *PDE4B* down‐regulation and *ADCY1*, *ADCY5*, and *ADCY6* up‐regulation could further demonstrate the remarkable reprogramming capacity of MFs.

The most recent gene to be investigated was *ADM*, which produces the peptide known as adrenomedullin, which is found in the brain, heart, and adrenal medulla. ADM interacts with cytoskeletal elements including microtubules to protect neurons, enhance cAMP synthesis, and mediate healthy neuron proliferation and morphological development.^63^, ^64^, ^65^ In mouse models of ischemia with infarct induction, a knock‐out of *ADM* at the neuronal level results in an increase in the volume of the infarcted area and aggravation of brain damage.^66^ However, a knock‐out of the same gene in endothelial cells results in a decrease of the infarcted area volume, and subsequent brain damage.^67^ Given the inconsistent nature of the literature, further investigation of our data regarding *ADM* down‐regulation in MFs is necessary.

We point out the following limitations of the study. A comparison with young fibroblasts, including meningeal and SFs, should be performed to confirm the results obtained with old fibroblasts. Indeed, these cells come from elderly individuals affected by neurological and vascular illnesses. The comparison of young and old fibroblasts, and a concomitant comparison of MFs and SFs from healthy and diseased subjects, could clarify some neurodegenerative processes affecting old people. Despite the low number of cases, it should be considered that the ability to get both types of fibroblasts from the same patient, with a similar age, gives this study a great advantage and enables more precise comparisons between the two cell types.

## 5 CONCLUSIONS

Our findings showed that MFs and SFs exhibit different characteristics in terms of morphology, metabolic activity, expression of protein markers, and transcriptome profile, demonstrating that they are deeply different cell types, identifying MFs as a key player in the CNS homeostasis as the non‐neural cell. In addition, the identification of nestin expression and up‐regulation of pathways involved in the cellular response to forskolin and cAMP metabolism in MFs opens the door for further investigation into MF's capacity to differentiate into mature neurons, which may result in new, individualised treatments for neurodegenerative illnesses. Finally, recent research on the existence of stem cell niches within the meninges shows how studying the meninges and the cells that reside there may produce unexpected results, greatly expanding the field of regenerative medicine.^68^, ^69^

## AUTHOR CONTRIBUTIONS

**VF, RRF, MB, and CC**: Conceptualization and project design. **VF, RRF, and ES**: Performed experiments. **VF, AD, VM, AG, and TEP**: Contributed to case selection. **VF, CP, VM, SG, and OP**: Data analysis and interpretation. **VF**: Original draft writing. **RRF, MB, AD, SG, AG, OP, and TEP**: Text revision. **OP, CC, and TEP**: Project supervisor. All authors have read and approved the final manuscript.

## FUNDING INFORMATION

This study was supported by “Fondo di Beneficenza Intesa Sanpaolo” (Italy); Project code: B/2022/0094” and by “Ricerca Corrente 2022‐2024, Italian Ministry of Health”.

## CONFLICT OF INTEREST STATEMENT

The authors declare no conflict of interest.

## PATIENT CONSENT STATEMENT

Informed Patient Consent has been obtained.

## Supporting information


**Supplementary Figure S1:** Meningeal and skin fibroblast isolation and sorting. (A) Meningeal explant after washing with 1X PBS. (B) Vessel removal from the meningeal explant and isolation of meningeal tissue. C Meningeal tissue fragmentation in 2–3 mm pieces. (D), (E) Pictures of meningeal (D) and skin (E) fragment plating. (F), (G) 10× magnification (EVOS XL Core Cell Imaging System microscope, Thermo Fisher Scientific, USA) of MFs (F) and SFs (G) at high confluency. (H), (I) Cell sorting protocol using Anti‐Fibroblasts MicroBeads (Miltenyi Biotec, Germany) and Magnetic Activated Cell Sorting (MACS®).

**Supplementary Figure S2:** Grow direction analysis. (A)–(D) Grow direction analysis were obtained using immunofluorescence images of β‐III‐tubulin labelling using Axio Imager 2 microscope with Axiocam Mrm camera (Zeiss, Germany) microscopy at 40× magnification (A for MFs–B for SFs) and directionality histograms related to MFs (C) and SFs (D). Maps and histograms were obtained using the Directionality plugin from ImageJ (NIH, USA, https://imagej.nih.gov)

**Table S1:** List of subjects recruited for this study.
**Table S2:** List of primers sequences.
**Table S3:** List of antibodies.

## Data Availability

The datasets generated and/or analyzed during the current study are available in the GEO repository, [GSE255684]. All other data generated or analyzed during this study are included in this published article and its additional information files.
